# Cancer incidence and digital information seeking in Germany: a retrospective observational study

**DOI:** 10.1038/s41598-024-60267-4

**Published:** 2024-05-03

**Authors:** Hannah Wecker, Daniel Maier, Stefanie Ziehfreund, Fabienne A. U. Fox, Ian Erhard, Jörg Janne Vehreschild, Alexander Zink

**Affiliations:** 1 Department of Dermatology and Allergy, TUM School of Medicine and Health, Technical University of Munich, Munich, Germany; 2 Medical Department 2 (Hematology/Oncology and Infectious Diseases), Center for Internal Medicine, Goethe University Frankfurt, University Hospital, Frankfurt am Main, Germany; 3 German Cancer Consortium (DKTK), Partner Site Frankfurt/Mainz and German Cancer Research Center (DKFZ), Heidelberg, Germany; 4 Department I for Internal Medicine, Faculty of Medicine and University Hospital Cologne, University of Cologne, Cologne, Germany; 5 German Centre for Infection Research (DZIF), Partner Site Bonn-Cologne, Cologne, Germany; 6 Division of Dermatology and Venereology, Department of Medicine Solna, Karolinska Institutet, Stockholm, Sweden

**Keywords:** Digital epidemiology, Infodemiology, Cancer prevention, Pan-cancer, Web search behavior, Cancer, Diseases, Health care, Oncology

## Abstract

Awareness is vital for cancer prevention. US studies show a strong link between web searches and cancer incidence. In Europe, the relationship remains unclear. This study characterizes regional and temporal relationships between cancer incidence and web searches and investigates the content of searches related to breast, cervical, colorectal, lung, prostate, and testicular cancer, brain tumors, and melanoma in Germany (July 2018–December 2019). Aggregate data from Google Ads Keyword Planner and national cancer registry data were analyzed. Spearman’s correlation coefficient (*r*_*S*_) examined associations between cancer incidence and web search, repeated measures correlation (*r*_*rm*_) assessed time trends and searches were qualitatively categorized. The frequency of malignancy-related web searches correlated with cancer incidence (*r*_*S*_ = 0.88, *P* = 0.007), e.g., breast cancer had more queries than the lower-incidence cervical cancer. Seasonally, incidence and searches followed similar patterns, peaking in spring and fall, except for melanoma. Correlations between entity incidence and searches (0.037 ≤ *r*_*rm*_ ≤ 0.208) varied regionally. Keywords mainly focused on *diagnosis*, *symptoms,* and *general information*, with variations between entities. In Germany, web searches correlated with regional and seasonal incidence, revealing differences between North/East and South/West. These insights may help improve prevention strategies by identifying regional needs and assessing impact of awareness campaigns.

## Introduction

Cancer is the second most frequent cause of death in Europe^1^. In Germany, more than 498,000 patients were diagnosed with cancer in 2018, with the most prevalent entities including breast, prostate, colorectal, lung, and skin cancer^2^. About 37% of these cancer diagnoses are estimated to have been preventable^3–6^.

Internet use is steadily rising alongside the rapid growth of information, including health-related materials. Individuals frequently turn to search engines to inform themselves about diseases and assess their own condition^7^. In particular, patients with a diagnosed disease and their care providers search for information about respective diagnoses, risk factors, associated symptoms, prognosis, and therapy options^8–13^. This behavior applies to cancer as well^14^. One survey reported that approximately two-thirds of cancer patients turn to the internet in search of cancer-related information^15^.

Web search engines record user search history and location. These data can be analyzed to evaluate web search patterns, create search profiles, and infer user interests^16–18^. Studies from the US have found associations between web search volume (SV), the number of search queries for a specific search term, and the incidence of various cancer types^19,20^. Moreover, disease-specific web searches have been observed to spike after disease prevention campaigns, especially with respect to the Breast Cancer Awareness Month^18,21–23^ and after media coverage of diseased celebrities^24–26^.

While this body of research suggests that SV could be used as a proxy for public disease awareness^18,19,21^, it must be highlighted that SV as a type of digital trace data^27^ cannot be equated with the complex construct of disease awareness^28,29^. However, individuals translate health-related information needs into web search requests with the intention to identify and access respective information^30,31^. Thus, web searches reflect the engagement between users and information sources and in this sense can be seen as “individual proxies for public disease awareness”^32,33^. Individual motivations of searches (mere interest-driven vs. problem-driven) or the quality of consecutively accessed information resources cannot be derived solely from SV.

In this sense, we consider SV as an economical and accessible data source to approximate public disease awareness that may help to plan prevention measures effectively.

However, to the best of our knowledge, the relationship between the incidence of various cancers and SV as proxy of public awareness has not yet been studied in Germany. The aim of this study was to characterize the spatiotemporal relationship between incidence and SV in Germany for 8 cancer entities: breast, prostate, colorectal, lung, cervical, and testicular cancer, melanoma, and brain tumors. Incidence data were provided by the German Center for Cancer Registry Data (German: *Zentrum für Krebsregisterdaten,* ZfKD), and SV was retrieved from Google’s Ads Keyword Planner. Specifically, we examined the incidence and SV for each of these cancers, assessed temporal and regional patterns, and investigated the association between cancer-specific incidence and SV. To identify themes of high public interest, we explored the content of the entity-specific web searches.

## Methods

### Data

**Figure 1:**
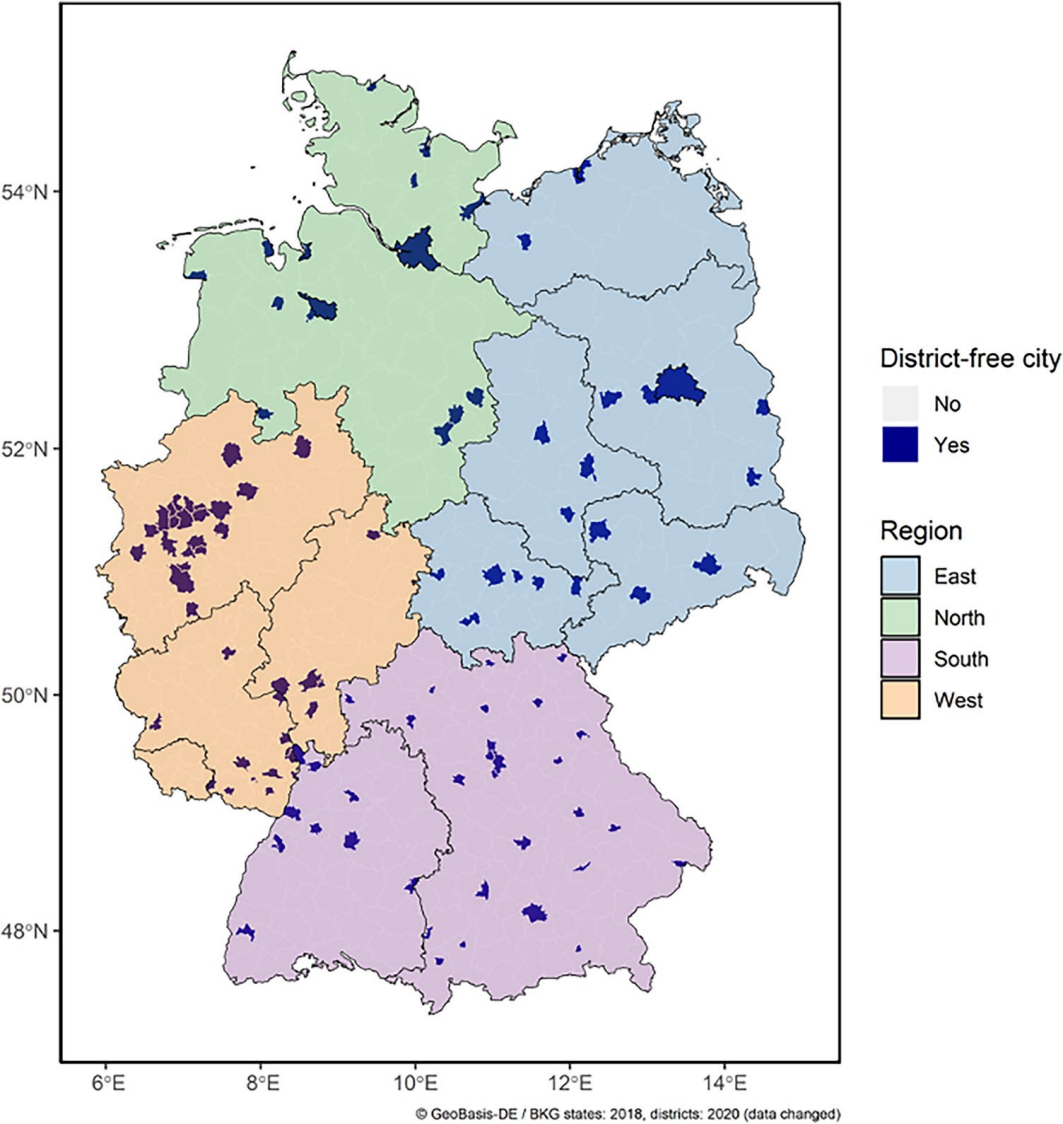
Map of Germany with all N = 107 district-free cities, with color-coded regions. In total, there were 15 district-free cities in the North, 19 in the East, 34 in the South, and 39 in the West. For visualization of the selected district-free cities and federal states of Germany, publicly available geospatial data based on the German Federal Agency for Cartography and Geodesy were obtained from Esri Germany.

This retrospective, observational study combined monthly data on cancer incidence and SV from July 2018 to December 2019 for district-free cities in Germany. District-free cities (German: *Kreisfreie Städte*) are units where the city proper is identical to the area of the administrative unit. We restricted our analysis to district-free cities, as they were the smallest spatial unit for which both cancer incidence and SV data were available and could be linked. With respect to the negative association of rural living on health care utilization^34^ and the heterogeneity of internet coverage in urban vs. rural areas^35^, limiting the analysis to district free cities aimed to avoid these potential confounders. Data from all district-free cities (N = 107) were investigated (Fig. 1). The total population of all district-free cities was 26.9 million inhabitants, representing about one-third of the German population^36^.

Based on previous findings that disease incidence, prevention campaigns, and disease-related survival could drive disease awareness^16,18–20,24,37^, we selected the above-mentioned cancer entities based on their respective incidence, survival, and prevention campaigns (high incidence: breast, prostate, colorectal, lung, and melanoma; low disease-related survival: lung cancer and brain tumor; prevention campaigns: breast, prostate, colorectal, cervical, and testicular cancer and melanoma).

### Cancer incidence data

Data on cancer incidence by district and month were provided on request by the ZfKD, at the Robert Koch Institute^38^. The ZfKD’s data collection procedure is detailed in^39^; details on the registry coverage can be found in^40^. Diagnoses were based on the International Classification of Diseases 10th revision (ICD-10, Supplementary Table S1). In addition, we extracted the month and year of diagnosis and the district area code of residence of each diagnosed person. We limited the data analysis to the period of July 2018 to December 2019 as congruent web search and registry data were only available for this period.

### Web search data

In Germany, Google is the most frequently used search engine with a market share above 90%^41^. SV data were, thus, based on queries to the Google search engine and its network partners and gathered via the Google Ads Keyword Planner^17,42,43^. For each search term entered, this tool provides a comprehensive list of keywords and phrases as well as the number of searches from a given geographic area in the past 48 months. To capture a broad range of search terms, we entered a medical and a lay term for each of the 8 above-mentioned malignancies (Supplementary Table S1). Data extraction was restricted to keywords and phrases in German and to web searches that could be traced back to one of the 107 district-free cities.

All identified keywords (k = 12,551) were manually reviewed. Keywords that were not directly linked to investigated diseases were excluded from further analysis (e.g., “antihormone therapy side effects”). For skin cancer-related web searches, we additionally excluded all keywords related to keratinocyte carcinoma (33.5%) and restricted the search terms to keywords related to melanoma.

### Data linkage

Cancer incidence and web search data were linked based on location and date, resulting in 18 monthly observations for each pair of cancer entity and district-free city. To ensure comparability between heterogeneously populated cities, we scaled incidence (Inc/100k) and SV (SV/100k) per 100,000 inhabitants. We extracted the population size of each city in 2019 from the database of the Federal Office for Building and Regional Planning (German: *Indikatoren und Karten zur Raum- und Stadtentwicklung;* INKAR)^36^. To gain insight into regional differences, the district-free cities were grouped by a common regional partition of Germany, roughly corresponding to cardinal directions. In total, there were 15 district-free cities in the North, 19 in the East, 34 in the South, and 39 in the West. For visualization of the selected district-free cities and federal states of Germany, publicly available geospatial data based on the German Federal Agency for Cartography and Geodesy were obtained from Esri Germany^44^.

To obtain access to the incidence data, the study protocol was handed in and approved by the scientific review board of the ZfKD. Institutional review board approval and informed consent were not required for web search data due to its non-disclosive nature and public availability.

### Statistical analysis

For each malignancy, we assessed both the time- and space-aggregated distributions of Inc/100k and SV/100k using median and interquartile range (IQR). To determine whether the incidence of malignancies is associated with SV, we computed Spearman’s correlation coefficient (*r*_*S*_) between the time- and space-aggregated totals of absolute incidence and SV.

To examine the temporal patterns of cancer-specific incidence and SV, we visualized space-aggregated Inc/100k and SV/100k with line plots across months. We calculated normalized means and Gaussian 95% confidence intervals (CI). Depending on the underlying distribution, regional differences of time-aggregated cancer Inc/100k and SV/100k were assessed with ANOVA, Welch-ANOVA, or Kruskal–Wallis tests. To test for differences between regions, we performed Tukey, Games-Howell, or Dunn’s post-hoc tests (*P*_*post-hoc*_) in follow-up analyses. *P* values of all post-hoc tests were corrected with the Bonferroni method. Before testing for regional differences using the respective methods, the assumption of approximate normality was assessed graphically and the homogeneity of variance was tested using Levene's test. To determine overall as well as region-specific associations between Inc/100k and SV/100k, repeated measures correlations (*r*_*rm*_) and corresponding 95% CIs were calculated for each cancer entity^45^. Additionally, the *P* values for the overall correlations were adjusted using Bonferroni correction and the region-specific correlations using Benjamini–Yekutieli correction, as their *P* values may be interdependent. For all analyses, two-sided tests were performed and the significance level was set to 0.05. Statistical analyses were performed using the statistical software R version 4.2.3 (R Core Team, 2021, Vienna, Austria).

The web search keywords were classified deductively and inductively and emerging topics were identified and discussed iteratively between the researchers (HW, DM, and SZ) until 15 final categories were determined: *diagnosis* (a diagnosis-indicating disease specification; e.g., “stage IV breast cancer”), *symptoms* (e.g., “weight loss colon cancer”), *treatment* (e.g., “skin cancer treatment”), *prognosis* (e.g., “glioblastoma life expectancy”), *risk factors/triggers* (e.g., “lung cancer smoking”)*, demographics* (e.g., “lung cancer women”), *comorbidity* (e.g., “Crohn’s disease colon cancer”), *consequences* (e.g., “impotence after prostate cancer”), *prevention* (e.g., “hpv pap smear cervical cancer”), *costs* (e.g., “surgery costs colon cancer”), *celebrities* (e.g., “Kylie Minogue breast cancer”), *general* information (e.g., “melanoma”), *media* (e.g., “brain tumor documentary”), *peer-community* (e.g., “lung cancer experience reports”), and *others* (e.g., “prostate cancer cycling”)*.* Finally, one researcher (HW) resolved conflicting keyword categorizations by different coders to ensure that each search term was exclusively assigned to one category. The content of malignancy-related web searches was analyzed descriptively by calculating the percentage of time- and space-aggregated SV/100k for each category within a malignancy.

## Results

### Descriptive analysis of cancer incidence and web search volume

In total, 126,350 inhabitants of German district-free cities were diagnosed with cancer between July 2018 and December 2019 (Table 1). During the same period, a total of 21,116,930 malignancy-related web searches were recorded in these district-free cities, after excluding 1,256 irrelevant keywords out of a total of 12,759 cancer-related German keywords and phrases.

**Table 1:** Malignancy-specific incidence and web search volume in total and time- and space-aggregated reported with median per 100,000 inhabitants including interquartile range between July 2018 and December 2019. The ranks of each entity’s total incidence and search volume are displayed in round brackets, from highest (1) to lowest (8).

Malignancy	Total incidence, n (rank)	Incidence per 100,000 inhabitants, median [IQR]	Total search volume, n (rank)	Search volume per 100,000 inhabitants, median [IQR]
Brain tumor	2891 (6)	0.40 [0.00; 0.92]	2,743,900 (5)	635.90 [525.13; 822.60]
Breast cancer	32,511 (1)	6.80 [5.06; 8.53]	4,314,110 (1)	1,054.90 [891.26; 1,352.41]
Cervical cancer	2115 (7)	0.00 [0.00; 0.65]	1,278,560 (7)	306.80 [246.94; 404.55]
Colorectal cancer	24,906 (4)	5.30 [3.94; 7.00]	2,499,140 (6)	607.30 [496.96; 759.83]
Lung cancer	27,121 (2)	5.40 [3.71; 7.48]	3,494,020 (2)	852.10 [707.91; 1,054.72]
Melanoma	9101 (5)	1.90 [1.00; 2.97]	3,280,220 (3)	763.20 [612.82; 974.52]
Prostate cancer	25,889 (3)	5.80 [4.24; 7.52]	2,770,460 (4)	691.80 [554.24; 898.67]
Testicular cancer	1816 (8)	0.0 [0.00; 0.60]	736,520 (8)	172.60 [135.53; 231.38]
Total	126,350		21,116,930	

*IQR* interquartile range.

Between July 2018 and December 2019, breast, prostate, lung, and colorectal cancer had the greatest median Inc/100k. Median SV/100k were highest for breast cancer, lung cancer, and melanoma. On the other end, we observed the lowest incidence rates and SV for cervical and testicular cancer (Table 1). Cancer incidence was strongly associated with SV as reflected by a Spearman’s correlation coefficient of *r*_*S*_ = 0.88 (*P* = 0.007).

### Temporal and spatial patterns of cancer incidence and search volume

**Figure 2:**
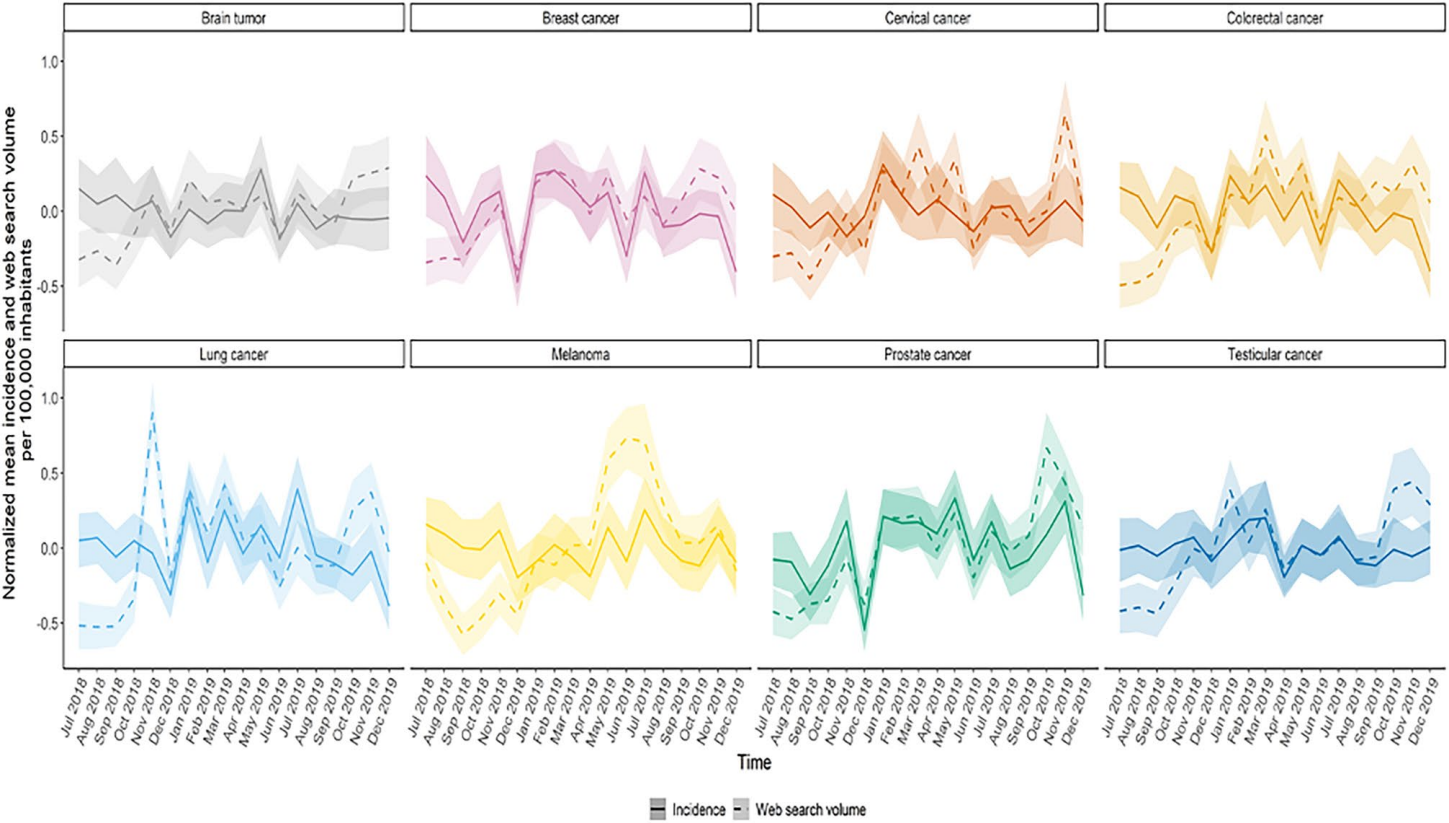
Malignancy-specific normalized mean and 95% confidence interval of space-aggregated incidence and search volume per 100,000 inhabitants across the months between July 2018 and December 2019.

For all malignancies, normalized mean Inc/100k and SV/100k showed similar patterns across months with marked decreases in June, August, September, and December (Fig. 2). However, the normalized mean SV/100k for melanoma increased substantially in the summer months.

**Figure 3:**
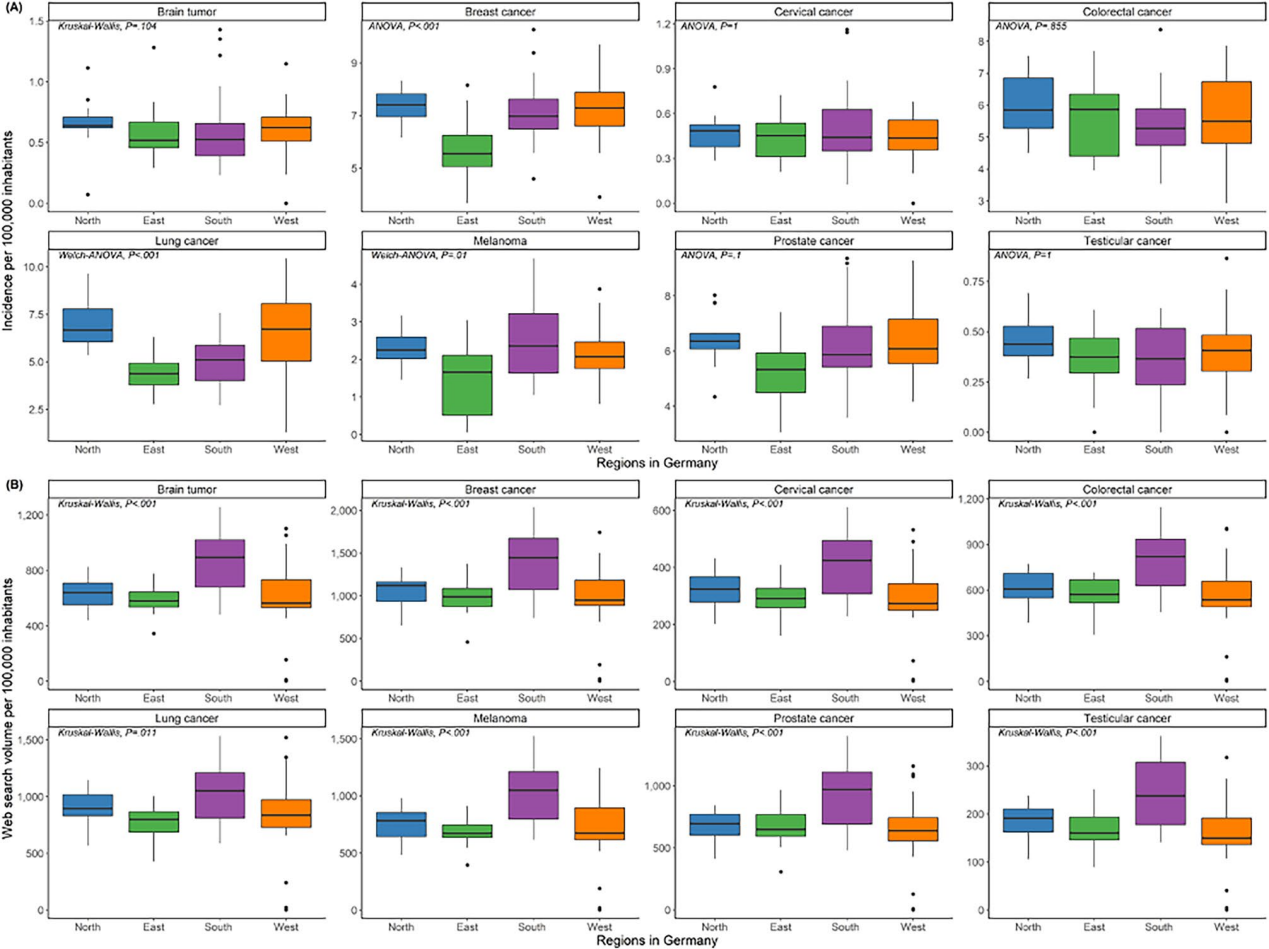
Malignancy-specific boxplots of cancer incidence (**A**) and search volume (**B**) per 100,000 inhabitants for each of the regions: North, East, South, and West Germany. The respective statistical methods and *P* values to assess differences between regions are reported.

Regional differences in Inc/100k were most pronounced for breast cancer, prostate cancer, and melanoma (Fig. 3), with the lowest incidence rates in the East compared to other regions (breast: *P*_*post-hoc*_ < 0.001; prostate: 0.03 ≤ *P*_*post-hoc*_ ≤ 0.04; melanoma: 0.003 ≤ *P*_*post-hoc*_ ≤ 0.03). We also found regional differences in the incidence of lung cancer (*P*_post-hoc_ < 0.001), though not consistently across all region pairs. A lower cancer incidence was recorded in Eastern Germany for 5 of 8 malignancies.

Differences in SV/100k between German regions were observed for the majority of malignancies. In particular, we found differences between Southern Germany and the other regions with a higher SV/100k for brain tumors (*P*_*post-hoc*_ ≤ 0.01), breast cancer (*P*_*post-hoc*_ ≤ 0.03), prostate cancer (*P*_*post-hoc*_ ≤ 0.02), and melanoma (*P*_*post-hoc*_ ≤ 0.02). SV/100k for lung cancer differed between the South and East (*P*_*post-hoc*_ = 0.002) and between the South and West (*P*_*post-hoc*_ = 0.02).

Comparing the temporal patterns per region (Supplementary Fig. S2), we observed similar trajectories of normalized mean Inc/100k and normalized mean SV/100k for most malignancies. For instance, we detected a similar pattern of cancer incidence and normalized search volume for prostate cancer in the West and South. Across all regions, we found seasonal variations with cancer Inc/100k and SV/100k decreasing in June, August, September, and December. Furthermore, search volume for lung cancer peaked in November 2018 in all regions.

### Association analysis

For all 8 entities, greater SV/100k was associated with higher Inc/100k (Table 2). Only for colorectal cancer was this positive correlation not significant (*r*_*rm*_ = 0.037, 95% CI: [− 0.009, 0.082]). The strongest correlations between Inc/100k and SV/100k were observed for testicular (*r*_*rm*_ = 0.208, [0.163, 0.251]), prostate (*r*_*rm*_ = 0.196, [0.152, 0.240]), and breast cancer (*r*_*rm*_ = 0.109, [0.064, 0.155]).

**Table 2:** Repeated measures correlation (r_rm_) between cancer incidence and search volume per 100,000 inhabitants, including 95% confidence interval (CI) and *P* values after correction for multiple testing overall and for each German region, cardinally classified into North, East, South, and West Germany.

Malignancy|Region	Repeated Measures Correlation, *r*_*rm*_ [95% CI], *P* value				
	Overall^a^	North^b^	East^b^	South^b^	West^b^
Brain tumor	0.095 [0.049, 0.140], *P* < 0.001	0.093 [− 0.029, 0.214], *P* = 0.735	0.131 [0.023, 0.237], *P* = 0.157	0.048 [− 0.034, 0.129], *P* = 1	0.167 [0.092, 0.240], *P* < 0.001
Breast cancer	0.109 [0.064, 0.155], *P* < 0.001	0.189 [0.068, 0.305], *P* = 0.034	0.108 [− 0.001, 0.214], *P* = 0.339	0.093 [0.011, 0.173], *P* = 0.207	0.111 [0.035, 0.186], *P* = 0.054
Cervical cancer	0.087 [0.042, 0.133], *P* = 0.002	0.129 [0.007, 0.248], *P* = 0.298	0.097 [− 0.012, 0.204], *P* = 0.475	0.084 [0.002, 0.164], *P* = 0.314	0.064 [− 0.012, 0.139], *P* = 0.566
Colorectal cancer	0.037 [− 0.009, 0.082], *P* = 0.949	0.074 [− 0.049, 0.195], *P* = 1	0.078 [− 0.031, 0.185], *P* = 0.840	0.014 [− 0.068, 0.095], *P* = 1	0.036 [− 0.041, 0.111], *P* = 1
Lung cancer	0.082 [0.037, 0.128], *P* = 0.004	0.168 [0.047, 0.285], *P* = 0.075	0.199 [0.092, 0.301], *P* = 0.005	− 0.002 [− 0.083, 0.080], *P* = 1	0.101 [0.025, 0.176], *P* = 0.092
Melanoma	0.070 [0.024, 0.116], *P* = 0.021	− 0.041 [− 0.162, 0.082], *P* = 1	0.132 [0.023, 0.238], *P* = 0.157	0.081 [0, 0.162], *P* = 0.339	0.071 [− 0.005, 0.146], *P* = 0.416
Prostate cancer	0.196 [0.152, 0.240], *P* < 0.001	0.173 [0.051, 0.289], *P* = 0.066	0.026 [− 0.083, 0.135], *P* = 1	0.206 [0.126, 0.283], *P* < 0.001	0.273 [0.201, 0.342], *P* < 0.001
Testicular cancer	0.208 [0.163, 0.251], *P* < 0.001	0.273 [0.156, 0.383], *P* < 0.001	0.297 [0.194, 0.393], *P* < 0.001	0.172 [0.092, 0.250], *P* = 0.001	0.172 [0.097, 0.245], *P* < 0.001

^a^Correction for multiple testing via Bonferroni correction.

^b^Correction for multiple testing via Benjamini–Yekutieli correction.

The associational strength between Inc/100k and SV/100k varied across German regions (Table 2). For most cancer entities (6 of 8), the North or East showed stronger correlations than the other regions. The direction of these correlations was fairly consistent: 30 of 32 region-entity pairs had a positive correlation between Inc/100k and SV/100k. Yet for about 72% of the region-entity combinations, correlation between Inc/100k and SV/100k were not significant.

### Search content analysis

**Figure 4:**
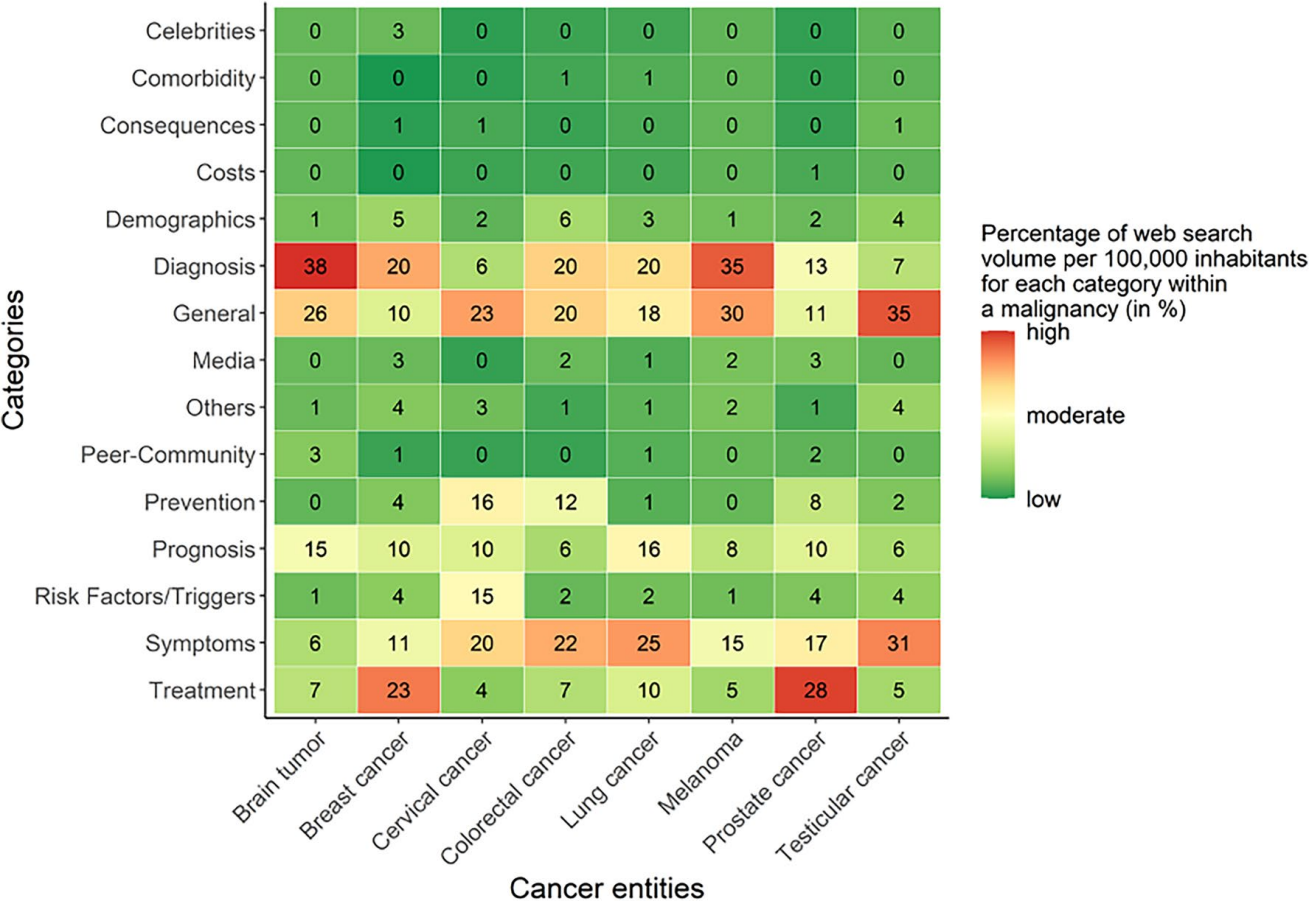
Percentage of malignancy-specific search volumes per 100,000 inhabitants for each search category. Keywords were classified deductively and inductively into 15 categories: diagnosis (a diagnosis-indicating disease specification; e.g., “stage IV breast cancer”), symptoms (e.g., “weight loss colon cancer”), treatment (e.g., “skin cancer treatment”), prognosis (e.g., “glioblastoma life expectancy”), risk factors/triggers (e.g., “lung cancer smoking”), demographics (e.g., “lung cancer women”), comorbidity (e.g., “Crohn’s disease colon cancer”), consequences (e.g., “impotence after prostate cancer”), prevention (e.g., “hpv pap smear cervical cancer”), costs (e.g., “surgery costs colon cancer”), celebrities (e.g., “Kylie Minogue breast cancer”), general information(e.g., “melanoma”), media (e.g., “brain tumor documentary”), peer-community (e.g., “lung cancer experience reports”), and others (e.g., “prostate cancer cycling”). Colors are only interpretable within one cancer entity. Percentages may not sum up to 100 due to rounding.

The content of web searches differed across malignancies (Fig. 4). For the majority of cancer entities, the most frequently queried keywords were related to cancer *diagnosis*, *symptoms*, and *general* information. These search categories were among the top 3 for 7 (*diagnosis*, *symptoms*) and 6 (*general* information) of the 8 entities, respectively. For brain tumors and lung cancer, searches related to *prognosis* were more frequent than in the other entities*.* The percentage of entity-specific web searches related to *prevention* was highest for cervical and colorectal cancer. For breast and prostate cancer, searches mainly focused on *treatment* options.

## Discussion

### Main results

In this study, we investigated spatiotemporal relationships between entity-associated SV and cancer incidence for 8 malignancies in Germany. Across all 8 entities, we observed that greater incidence rates were associated with higher SV. Specifically, more highly incident cancer entities, such as breast and lung cancer, were more frequently queried than rarer entities, such as testicular and cervical cancer.

The trajectory of incidence and SV followed a seasonal pattern: incidence and SV declined in the summer (July and August 2019) and winter months (December 2018, December 2019). A study in Sweden observed a similar pattern and attributed reduced incidence rates to lower detection rates during holiday seasons^46^. While we cannot draw causal inferences from our data, we may hypothesize that reduced incidences in Germany may be due to restricted opening times of doctors’ offices and less staffing during the holiday season.

In line with previous studies, we found higher incidence rates and SV for melanoma during the summer^11,42,43^. We expected to observe this pattern as solar ultraviolet radiation is a major risk factor for melanoma and awareness campaigns for skin cancer are particularly active in the summer^42^. Additionally, we note that elevated SV coincided with prevention campaigns (e.g., breast cancer awareness month in October, lung cancer awareness month in November, world cancer day in February)^21–23^ and media reports. For example, a spike in lung cancer SV could be observed in November 2018, the same month as a highly publicized death of a German celebrity to lung cancer^47^.

Across German regions, a lower cancer incidence has been indicated in Eastern Germany for most malignancies. This is partly in line with a previous study that reported a lower cancer incidence in women living in Eastern Germany compared to women in Western Germany, though the opposite trend was found in men^48^. Furthermore, Vogt et al. found higher screening rates for various cancers in districts in Eastern Germany than districts in the West^49^, which may result in lower incidence rates due to early detection of precancerous lesions^50^. We also found regional differences in web searches, with higher SV for all malignancies in Southern Germany. Compared to the other regions, Southern Germany has the youngest inhabitants^36^. Younger individuals have been found to search more frequently for health information on the internet than older individuals^8,51^. Thus, differences in SV across regions may be at least partially attributable to demographic differences. Further research is required to study how regional differences in demographic characteristics, such as sex, age, and socioeconomic status, as well as the accessibility and distribution of medical care facilities, are linked to SV and cancer incidence.

We observed statistically significant small to moderate associations between cancer Inc/100k and SV/100k for all malignancies except colorectal cancer. For these entities, increases in SV/100k were associated with rising Inc/100k. Importantly, the strength of this correlation differed across regions. For breast and cervical cancer, we found the strongest correlation between cancer incidence and SV in Northern Germany, whereas the association for melanoma, colorectal, lung, and testicular cancer were strongest in Eastern Germany. While we observe a link between cancer incidence and SV, other factors such as access to close-by health care facilities, health promotion (media) campaigns etc. certainly play a major role in steering public awareness. Further research is needed to investigate the causes of regional differences and the interplay of online health seeking behavior with health information promoting factors.

Web search content differed across cancer entities. Comparatively, web searches related to brain tumors and lung cancer focused relatively more often on disease prognosis. This may be due to their low disease-related survival probabilities. On the other hand, web searches related to entities with multiple available therapy options—such as breast and prostate cancer—particularly targeted information about treatments^10–12^. In addition, web searches related to cervical and colorectal cancer also focused on preventive measures, such as screening^52^.

### Limitations

Our analysis is based on observational, aggregated data at a population level. Thus, reliable statements about causal relationships between cancer incidence and SV cannot be derived.

Cancer incidence data were provided by the ZfKD, which brings together cancer registry data at the national level and can be regarded as a source of reliable, standardized data. SV in contrast is a type of digital trace data that can only serve as a surrogate measure for disease awareness.

Our results may not generalize to all German regions and cancer entities:

We used data from Germany’s district-free cities (N = 107) and did not include smaller urban and rural areas. Thus, our findings may not be representative of the entire German population. We also restricted our analysis to 8 cancer entities, which were selected based on incidence, prevention measures (i.e., campaigns), and survival. Across regions and entities, we observed differing strengths of association between web searches and cancer incidence. The observed patterns may not extend to other cancer entities.

Further, SV was based on German search terms only. Thus, we cannot infer information on the search behavior of non-German speakers. Additionally, our inferences rely on German search results being representative of the search behavior of German-speaking inhabitants of the cities investigated.

Due to the restricted overlap of available SV and cancer incidence data, we could only analyze time series data with 18 monthly time steps. This precluded the application of time series regression and the robustness of seasonal effects analysis. Rigorous testing of the observed declines in cancer incidence during the German holiday months and the seasonal elevation of melanoma awareness in summer as reflected in SV are still needed.

Previous research finds that young, female, and highly educated adults are more likely to use the internet to search for health information than older, male, and less educated individuals^16,30,51,53^. We cannot infer the characteristics of the individuals, on whom the web search data in this study was based. However, we speculate that information searching behavior of older individuals, who are typically at a higher risk for cancer, is underrepresented in this study.

Epidemiological studies have shown that lung and colorectal cancer incidence and mortality increase with higher deprivation of living area^54,55^. Area deprivation also is negatively associated with access to health care^34^. Thus, living area deprivation could be a unobserved confounder in our study, that future research should address.

### Comparison with prior work

Our results contribute to an existing body of similar research from other geographical contexts. While studies in the US^19,37^ and China^16^ have observed strongly positive correlations between cancer incidence and SV, we only found low to moderate associations, comparable to those reported by Phillips et al.^18^. This may be attributable to our methodological approach of narrowing the geographical scope to district-free cities instead of the state level. We examined the association between SV and cancer incidence across months with repeated measures correlations. Interestingly, after adjusting for the measurement period, Phillips et al.^18^ also reported substantially lower correlation coefficients.

Online information-seeking behavior on multiple cancer entities has been studied through the lens of Google data in and across various countries—in particular in the US^18,20,37^, but also in China^16^ and Canada^56^. Prior research on disease-related and web-based information-seeking predominantly used Google Trends^18–20,24,25,37,56,57^ instead of Google Ads Keyword Planner^42,43^. We considered both data sources but decided on the latter, as it provides more fine-grained information, including geographical units and absolute counts instead of relative frequency abstractions. Moreover, Google Ads Keyword Planner allows for the analysis of keywords and phrases related to search terms, which provides more detailed insight into the public's interests, unmet needs, and awareness of different types of cancer^17^. Thus, to the best of our knowledge, this study is not only the first to examine the association between SV and multiple cancer entities, but also the first to assess the relationship at this level of geographical detail.

## Conclusions

We report moderate but consistent positive associations between cancer incidence and SV for a set of cancer entities in German district-free cities. Examining the relationship between SV—as a proxy of public awareness—and incidence—as one of its key drivers—reveals regions with higher disparity between cancer incidence and SV. Such disparities could signify socially-deprived areas with unmet information needs^24^ and could be of use for planning future prevention measures.

Our investigation is a first step toward using web search information to understand German public awareness about cancer. Further research is needed to describe more precisely the relationship between public awareness and web search behavior. This will likely require the development and empirical validation of models that describe the interplay of social dynamics, perception, and group and individual actions.

### Supplementary Information

Supplementary Information.

## Data Availability

The data that support the findings of this study are available from the corresponding author upon reasonable request.
