# Supplementary material for: Cancer incidence and digital information seeking in Germany: a retrospective observational study
Source: Sci Rep. 2024 May 3;14:10184. doi: 10.1038/s41598-024-60267-4 (PMC11068859; doi:10.1038/s41598-024-60267-4)

Table S1 The cancer entities studied with the corresponding International Classification of Diseases (ICD-10) and the search terms used, both medical and lay terms, to generate web search data.

| **Cancer entity (ICD-10 code)** | **Medical term** | **Lay term** |
| --- | --- | --- |
|  |  |  |
| **Brain (C71-C72)** | Glioma (German: “Gliom”) | Brain tumor (German: “Hirntumor”) |
| **Mamma (C50)** | Mamma carcinoma (German: “Mammakarzinom”) | Breast cancer (German: “Brustkrebs”) |
| **Cervix (C53)** | Cervix carcinoma (German: “Zervixkarzinom”) | Cervical cancer (German: “Gebärmutterhalskrebs”) |
| **Colon and rectum (C18-C20)** | Colon carcinoma, rectum carcinoma (German: “Kolonkarzinom”,  “Rektumkarzinom”) | Colon cancer (German: “Darmkrebs“) |
| **Lung (C34)** | Bronchial carcinoma (German: “Bronchialkarzinom”) | Lung cancer (German: “Lungenkrebs”) |
| **Prostate (C61)** | Prostate carcinoma (German: “Prostatakarzinom”) | Prostate cancer (German: “Prostatakrebs”) |
| **Melanoma (C43)** | Melanoma (German: “Melanom”) | Skin cancer (German: “schwarzer Hautkrebs”) |
| **Testis (C62)** | Testicular carcinoma (German: “Hodenkarzinom”) | Testicular cancer (German: “Hodenkrebs”) |

Figure S2 Temporal patterns of normalized mean incidence and search volume per 100,000 inhabitants between July 2018 and December 2019 with 95% confidence interval for each malignancy and German region, which were cardinally classified into North, East, South, and West Germany.


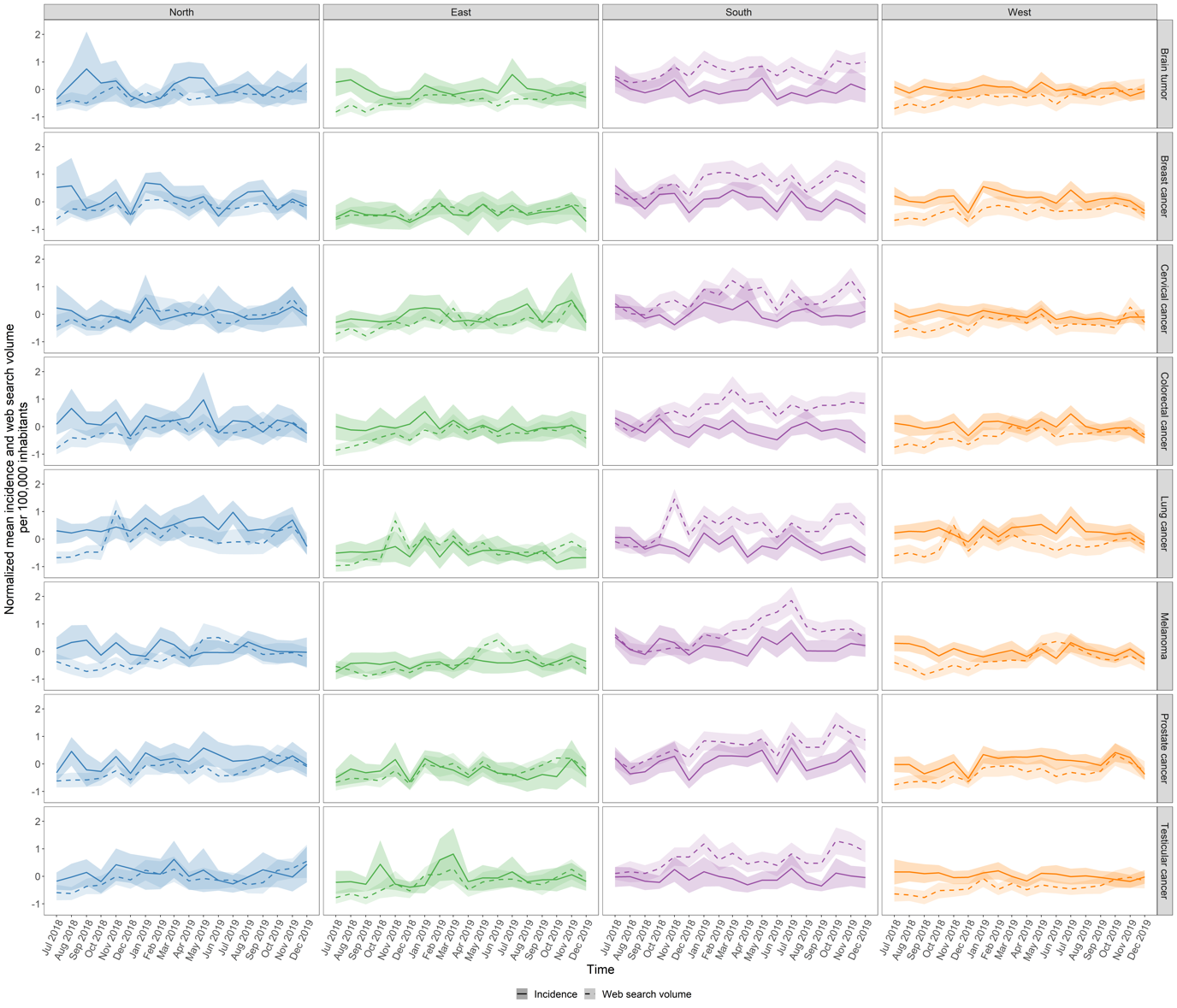

Supplement: Supplementary file 1 — Supplementary Information. [file 41598_2024_60267_MOESM1_ESM.docx]
